# Harnessing Non-Thermal Plasma to Supercharge Recovery in Abdominal Surgeries: A Pilot Study

**DOI:** 10.3390/jcm13020408

**Published:** 2024-01-11

**Authors:** Benjamín G. Rodríguez-Méndez, Régulo López-Callejas, Antonio Mercado-Cabrera, Rosendo Peña-Eguiluz, Raúl Valencia-Alvarado, Mario Betancourt-Ángeles, Guillermo Berrones-Stringel, César Jaramillo-Martínez

**Affiliations:** 1 Plasma Physics Laboratory, Instituto Nacional de Investigaciones Nucleares, Carretera México-Toluca S/N, La Marquesa, Ocoyoacac 52750, Mexico; benjamin.rodriguez@inin.gob.mx (B.G.R.-M.); regulo.lopez@inin.gob.mx (R.L.-C.); rosendo.eguiluz@inin.gob.mx (R.P.-E.); raul.valencia@inin.gob.mx (R.V.-A.); 2 Medical Center ISSEMyM Toluca, Av. Baja velocidad 284 km. 57.5, San Jerónimo Chicahualco, Metepec 52170, Mexico

**Keywords:** non-thermal plasma, surgical wound, therapy, wound healing

## Abstract

(1) Background: This study aims to evaluate the efficacy and safety of non-thermal plasma (NTP) therapy in accelerating wound healing in patients who have undergone laparoscopic and open surgeries. (2) Methods: NTP was applied using a needle-type reactor with an irradiance of 0.5 W/cm^2^ on the surgical wounds of fifty patients after obtaining informed consent. Three NTP treatments, each lasting three minutes, were administered hourly. (3) Results: The pilot study showed that NTP-treated surgical wounds healed completely without any signs of infection, dehiscence, pain, or itching. Notably, patients reported minimal pain after the NTP treatment. Visual assessments conducted twenty-four hours after surgery revealed no redness or fluid discharge. Comparisons with traditionally sutured wounds indicated that NTP-treated wounds healed at a rate equivalent to seven days. (4) Conclusions: The application of NTP in laparoscopic and open wounds proved safe and effective, expediting the wound healing process and eliminating clinical risks post-surgery. Significantly, NTP facilitated a healing rate within twenty-four hours, equivalent to seven days for suture-treated wounds, significantly reducing the hospitalization time to a single day. These findings highlight the potential of NTP to be a transformative approach for promoting postoperative recovery.

## 1. Introduction

The emergence of laparoscopic surgery has transformed the realm of surgical procedures, presenting a minimally invasive approach that consistently produces outstanding results and markedly enhances the overall quality of life for patients when compared to conventional open surgeries [1,2]. This advancement is underscored by the notable decrease in surgical site infections associated with laparoscopic procedures [1,2]. Nevertheless, the preference for open surgery persists in certain cases, emphasizing the necessity of a nuanced consideration of various factors in surgical decision-making [3].

Within the diverse spectrum of laparoscopic abdominal surgeries, ranging from cholecystectomies to complex interventions like low anterior resections and fundoplications, the overarching goal remains the restoration of compromised tissue integrity [4,5,6,7,8,9]. Notably, the evolution of some procedures to outpatient settings has yielded substantial benefits in terms of patient recovery and healthcare resource optimization [10,11,12].

The intricate wound healing process, crucial for postoperative well-being, unfolds through distinct phases influenced by many variables [13,14]. Among these factors, infection and wound management play pivotal roles and are particularly pertinent to the focus of this study. Postoperative infections not only complicate wound closure but also pose serious threats to patients’ quality of life, with potentially life-threatening consequences in severe cases [15].

Physical plasma is considered the fourth state of matter due to its high energy and ability to manipulate magnetic and electric fields [16]. It can be generated from gases using high voltages, magnetic fields, or intense electrical radiation [17]. A possible classification of physical plasma is as follows. Thermal plasmas are in thermodynamic equilibrium at high temperatures (like in the Sun) [18,19]. Non-thermal plasmas have highly energetic electrons and a much higher temperature than neutral ions and atoms [20,21]. Low-density plasmas have a reduced number of particles and allow the formation of complex structures [22,23]. High-density plasmas have many charged particles and can have complex behaviors [24,25].

In particular, in the non-thermal plasma (NTP), also known as cold atmospheric plasma (CAP), the electrons are highly energized, but at the same time, ions and atoms are maintained at temperatures close to room temperature [26,27]. This plasma generates reactive species essential in various industrial, medical, and environmental applications [28,29,30,31]. Reactive oxygen and nitrogen species are generated by this type of plasma, and they have antimicrobial properties and are used in surface disinfection and sterilization of medical instruments [28,32]. Likewise, these reactive species and UV radiation act as stimulants for accelerating blood coagulation mechanisms [33,34].

To address the urgent requirement for inventive methods to enhance the wound healing process, NTP therapy is emerging as a promising solution in healthcare. The production of reactive species during NTP therapy can facilitate cell proliferation and tissue migration, stimulate blood vessel formation, and prevent infections [35,36,37,38,39,40,41]. These species also have anti-inflammatory and analgesic properties [42,43].

This study bridges the translational gap, presenting a clinical investigation into applying NTP to assess its efficacy, safety, and potential for the acceleration of wound healing in patients undergoing either laparoscopic or open surgery. The findings promise to refine our understanding of innovative therapeutic modalities in surgical recovery, providing valuable insights into the intersection of advanced surgical techniques and cutting-edge wound healing technologies.

## 2. Methods and Procedure

### 2.1. Set-Up Description

A plasma needle-type reactor, previously detailed in comprehensive studies [35,44], was the foundation for the treatments explored in this investigation. The reactor operated within a helium gas atmosphere, maintaining a controlled flow of 0.5 LPM (liters per minute). The plasma needle was energized by an RF generator at 13.56 MHz, delivering a fixed power of 20 W. Consequently, an irradiance of 0.5 W/cm^2^ was applied to the wound, significantly below the 4 W/cm^2^ threshold. This deliberate limitation ensures the absence of any biological risk from non-ionizing radiation exposure to the patient’s tissues, adhering to safety standards outlined by the International Commission on Non-Ionizing Radiation Protection (ICNIRP) [45].

**Figure 1:**
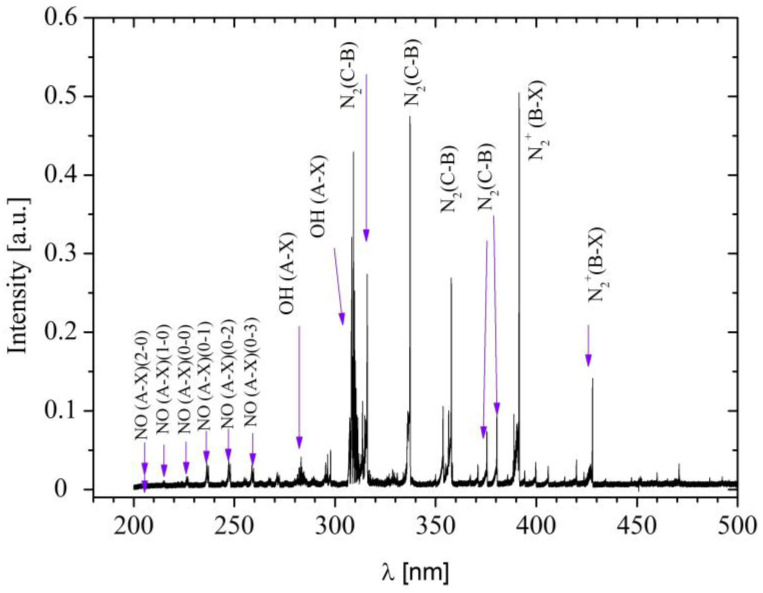
Optical emission spectra from needle-type plasma.

To discern the reactive plasma species generated by the NTP, optical emission spectroscopy (OES) was employed. The emission spectrum was captured by positioning an optical fiber end 5 mm from the plasma reactor’s outlet tip. Figure 1 depicts the UV region’s optical emission spectrum from plasma, utilizing helium as the working gas. The spectrum predominantly comprises molecular bands emitting light from NO, OH, N_2_, and N_2_^+^ systems.

Specifically, Figure 1 delineates the NOγ (A-X) system, arising from the dissociation of the surrounding air, with subsequent O and N atoms forming the NO molecule. The OH(A-X) system is attributed to the moisture content present in the air. Numerous N_2_(C-B) bands also manifest in the 300–400 nm region. The robust presence of the N_2_^+^(B-X) system at 391.4 nm and ~428 nm is noteworthy, and it is justified by using helium as the process gas. The energy required for N_2_ ionization (15.6 eV) is facilitated by excited states of He, which can possess energies up to 22 eV. Chemical species like NO and OH, identified in this analysis, hold paramount importance in biological and medical applications due to their pronounced reactivity [35,46,47].

Given the specific characteristics of the NTP generated and its application as a surface treatment, it is pertinent to highlight that the maximum depth of its impact on tissue within established timeframes is constrained to 1 mm [16].

### 2.2. Non-Thermal Plasma (NTP) Application Method

**Figure 2:**
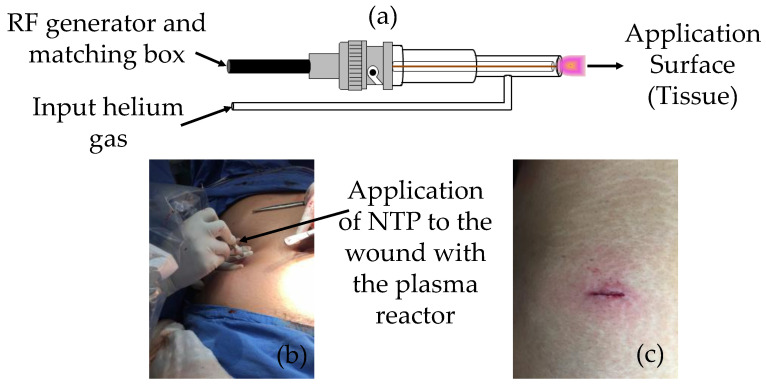
Device used for NTP treatment: (**a**) schematic diagram of the plasma reactor; (**b**) application of the NTP in the surgical wound; (**c**) view of the wound after applying NTP.

The applied NTP therapy method involved directing excited and reactive chemical species generated by helium and surrounding atmospheric plasma from a plasma reactor onto the targeted tissue. The device’s tip was strategically positioned approximately 5 mm from the wound, with deliberate, slow, and circular movements executed across the treatment area [48]. A detailed description of the treatment process with the NTP is presented in detail in Section 2.4. Figure 2 shows the device used.

### 2.3. Patient Selection and Protocol Approval

Following the approval of the medical protocol by the Health Research and Research Ethics Committees of the ISSEMyM Medical Center, a comprehensive framework was established to employ NTP for enhancing the healing process of abdominal surgical wounds conducted under general anesthesia. This study involved a meticulously calculated sample size of 50 patients, determined through sample calculation with a confidence level of 0.95 and a prevalence of 0.5 [49].

#### 2.3.1. Inclusion and Exclusion Criteria

Inclusion criteria encompassed individuals over 18 years of age, irrespective of gender, who, upon admission, provided written informed consent and underwent initial treatment at the ISSEMyM Medical Center. The patients included those undergoing laparoscopic and open techniques for inguinal hernia, fundoplication, and cholecystectomy.

Exclusion criteria comprised patients with pre-existing pathologies, such as kidney disease, liver disease, heart disease, lung disease, psychiatric disorders, arterial hypertension, a history of seizures associated with infectious processes, and those receiving medications with documented side effects on the central nervous system. Additionally, patients who declined to participate in this study were excluded.

#### 2.3.2. Informed Consent and Standardized Procedures

After the pre-anesthetic check-up, patients were presented with the protocol, and those who agreed provided written, validated, and informed consent. Treatment procedures and surgical techniques were standardized among participating surgeons before initiating the protocol. Only specialists with expertise in laparoscopic surgery conducted the procedures. Throughout the study period, fifty patients voluntarily participated in this study.

### 2.4. Experimental Procedure

The experimental protocol, conducted with the informed consent of fifty patients undergoing laparoscopic surgery wounds treated with NTP, unfolded through a meticulous and standardized sequence:

- Skin decontamination: the skin surrounding laparoscopic surgery wounds underwent thorough decontamination using a cleansing cream;
- Random wound selection: one wound was randomly selected for NTP therapy from the laparoscopic surgery wounds;
- Suturing: a synthetic absorbable polyglycolic acid suture, with sizes 1-0 and 3-0, was meticulously placed on the deep fascia and superficial fascia, respectively;
- Bleeding verification: rigorous verification for bleeding was conducted, with immediate cleaning of the area performed if any bleeding was identified;
- First NTP application: this treatment commenced in the operating room, with the first application lasting three minutes, followed by immediate photographic documentation;
- Second NTP application: this treatment occurred in the recovery room one hour after the first, again lasting for a three-minute duration and undergoing immediate photographic capture;
- Third NTP application: in this case, it was administered one hour after the second application, maintaining the three-minute duration, and another photograph was acquired promptly;
- Standardized photography: all photographs were consistently acquired using the same camera at pre-determined intervals;
- Postoperative verification: twenty-four hours post-surgery, the surgeon employed thumb and index fingers to verify the absence of dehiscence in both laparoscopic and open incisions, and a corresponding photograph was acquired;
- Follow-up at seven days: patients were scheduled for a follow-up appointment seven days after the surgery to assess any potential inconveniences arising from the NTP procedure.

This meticulously structured experimental procedure ensured standardized and comprehensive data collection, contributing to the robustness of this study’s findings. The careful sequence of interventions and assessments aimed to provide a detailed understanding of the effects of NTP on wound healing, fostering reliability and reproducibility in this study’s outcomes.

### 2.5. Laparoscopic and Open Surgery Procedures

Before surgery, the patient received preoperative care. Later, the patient was placed in a dorsal decubitus position on the surgery table in the operating room. General anesthesia or subdural block was applied, depending on the surgery. Sterile fields were established, and asepsis and antisepsis of the abdominal region were performed.

A trans-umbilical or supra-umbilical skin incision was made to access the abdominal cavity via laparoscopy. The abdominal wall was taken with Backhaus clamps, and field clamps were inserted. Subsequently, a 10–12 mm trocar was inserted with an activated punch. Once the aponeurosis was reached, the abdominal wall was elevated, and the aponeurosis of the more significant rectus muscle was transferred. With the laparoscopy equipment installed, namely lens, CO_2_ gas, and an electrocautery device, an operative field was created via insufflation with CO_2_ gas up to a pressure of 12 mm Hg (1600 Pa), the laparoscopic lens was inserted, and diagnostic laparoscopy was performed. Subsequently, the remaining trocars were placed under direct vision (generally between 3 and 5), with 5- or 12-millimeter measurements. Their placement formed a triangle to facilitate the mobilization of instruments through the operative field. They had to be positioned at least 8 cm apart to avoid unnecessary obstructions between the devices.

Open surgeries were performed using traditional procedures. The surgeon made an incision in the abdominal cavity near the affected organ, performed the corresponding repair, and closed the weakened abdominal muscles with a suture. Sometimes, they also placed a surgical mesh to strengthen the abdominal wall, and the incisions were sutured to close them.

### 2.6. Clinical Evaluation in Surgical Wound Healing

The evolution of the healing of the surgical wound was visually verified, observing its maturation and highlighting its color to detect the existence of edema or infection. The day after the surgery (approximately 24 h later), tension was applied around the wound lips to check that they were well adhered to; that is, the wound closure was checked. Likewise, a significant fact considered when evaluating the wound was the pain reported by the patient.

After the surgery and during the time in which the patient rested in the recovery room, we verified the following parameters:

- The amount of bleeding in the surgical wound;
- Wound closure time;
- Characteristics of healing;
- Pain at the surgical wound;
- Itching at the surgical wound;
- Adverse reactions such as wound infection or dehiscence.

The above parameters were also evaluated 24 h after surgery and three days after surgery. The patients from both surgeries were scheduled for wound revision and clinical evaluation. Surgical wound pain measurement was one of the endpoints for NTP therapy. We used the visual analog scale (VAS) since it is a relatively simple and effective instrument [50,51,52]. The scale values were “0” when there was no pain, a scale between 1 and 3 for mild wound pain, between 4 and 6 for moderate pain, and between 7 and 10 for severe pain.

## 3. Results

**Figure 3:**
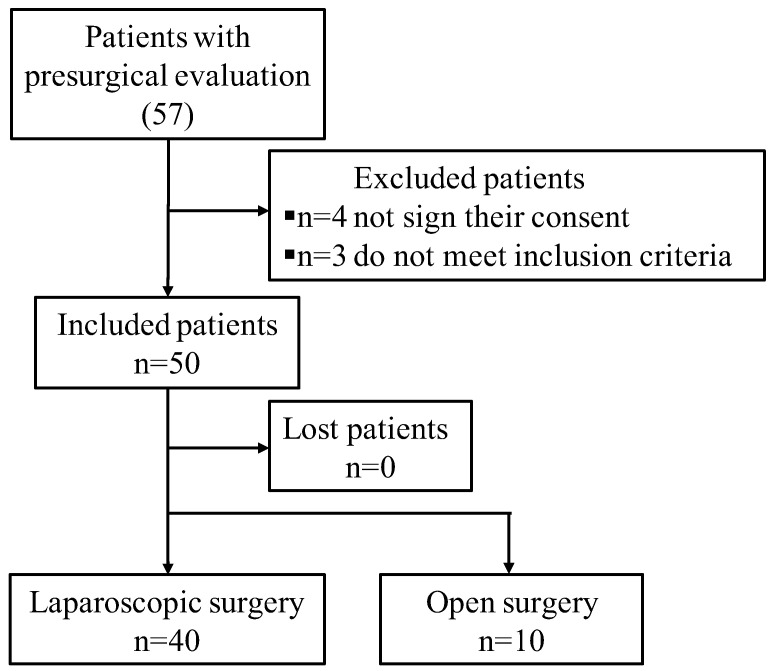
Patients recruited.

Figure 3 shows the flowchart through which the patients were selected for operations and to whom the non-thermal plasma was applied after the surgery.

The surgeries conducted on the fifty patients comprised the following procedures:

- Fourteen laparoscopic cholecystectomy surgeries utilizing four trocars (refer to Table 1);
- Eleven laparoscopic fundoplications, employing five trocars (refer to Table 1);
- Three laparoscopic appendectomies were performed using three trocars (refer to Table 1);
- Twenty-two inguinal plasties, with twelve performed laparoscopically using three trocars and ten performed as open surgeries (refer to Table 2).

The surgical procedures, encompassing both laparoscopic and open techniques, were performed on a cohort of fifty patients, and a detailed breakdown of these interventions is presented in Table 2, which meticulously describes the various surgical approaches employed, shedding light on the specific methodologies and techniques used in each case. By providing a comprehensive overview of laparoscopic and open surgeries, the table served as a valuable reference, offering information on the various surgical strategies implemented in this study. This detailed information was intended to contribute to a better understanding of the procedural nuances and outcomes associated with these different surgical modalities in the context of our patient population.

From a cohort of fifty patients treated with NTP, the initial thirty underwent the procedure described in Section 2.4. Observations revealed that laparoscopic lesions were closed immediately after the third NTP application, as outlined in the protocol. It is note-worthy that slight irritation was observed around the treated wounds, which were considered normal and attributed to the manipulation of the trocars used in this type of surgery.

A significant outcome achieved with NTP application was the absence of bleeding in the wounds. Additionally, the healing process accelerated, and the wound lips were observed to have been fully joined after completing the proposed procedure. Patients who were already conscious in the recovery room reported no pain in the NTP-treated wounds. Furthermore, none of the treated cases exhibited symptoms of infection, in contrast to conventionally treated injuries.

**Figure 4:**
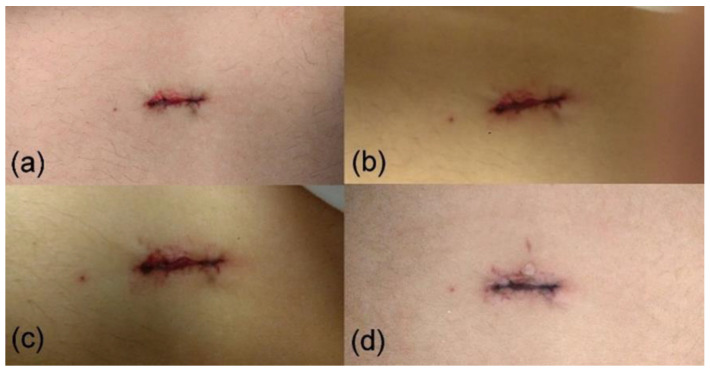
Evolution of closure of a 22 mm wound after (**a**) the first treatment in the operating room immediately after the surgery, (**b**) the second treatment one hour after surgery, (**c**) the third treatment two hours after surgery, and (**d**) 24 h post-surgery.

Figure 4a–d illustrates an example of a wound treated with NTP, capturing its temporal evolution at 1, 2, 3, and 24 h post-surgical intervention. Following NTP application, the surgical wound at the selected port displayed well-aligned edges, with no evidence of injured tissue. Macroscopically, it exhibited a more rapid healing process than conventional treatment methods.

Overall, the outcomes of employing NTP therapy in laparoscopic surgeries were highly satisfactory. No adverse effects were observed in the surgeries, prompting the medical staff to apply the treatment exclusively in the operating room.

**Figure 5:**
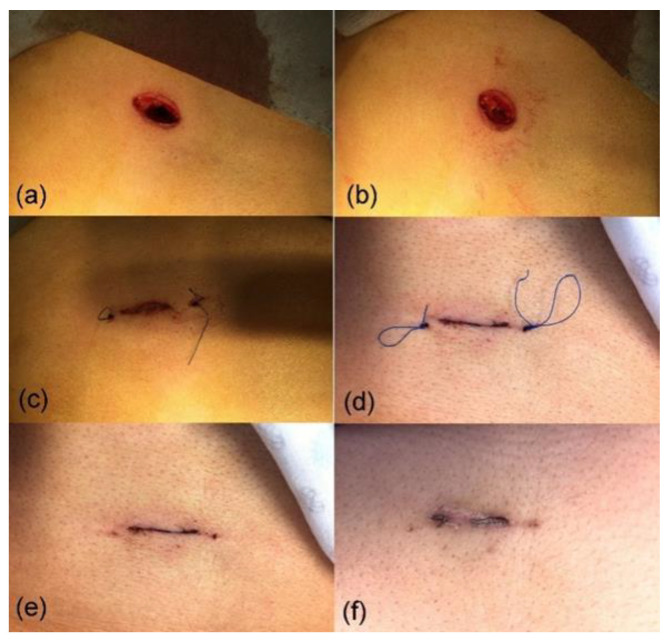
Development in the closure of two 19 mm long wounds, showing its evolution after (**a**) surgery, (**b**) the first treatment with NTP was applied before saturation of the peritoneum, (**c**) being subdermal sutured and receiving the second treatment, (**d**) 16 h post-surgery with surgical knots, (**e**) 16 h post-surgery without suture, and (**f**) three days post-treatment with NTP.

During surgery, before suturing the peritoneum (Figure 5b), NTP therapy was applied. This led to evident blood coagulation and tissue regeneration in the wound bed. The superficial fascia was then sutured (Figure 5c) with sub-dermal stitches using a polyglycolic acid suture. Following this, NTP therapy was applied again for three minutes on the wound. A transparent polyurethane bandage covered the wound after each NTP treatment.

The efficacy of NTP therapy was observed in the accelerated healing process of the superficial wound. Surgeons noted that tissue recovery was comparable to the typical wound closure time of seven to ten days. Additionally, none of the NTP-treated wounds exhibited infection or bleeding problems. The therapy also promoted accelerated coagulation and facilitated the union of wound lips during the second treatment application.

Furthermore, patients have reported a significant reduction in pain in NTP-treated wounds compared to non-treated wounds. This suggests that NTP therapy holds potential benefits in post-surgery pain management.

**Figure 6:**
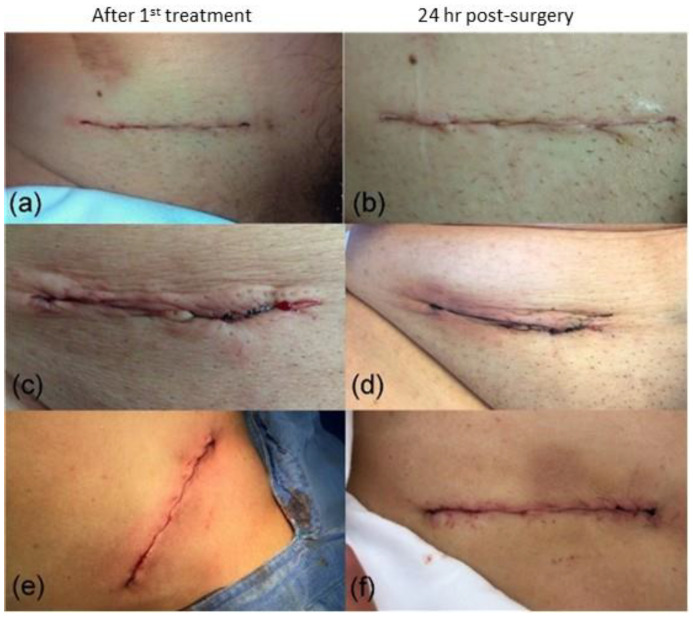
Evolution of wound closure in three different patients with open abdominal surgeries after applying the NTP. Their lengths were (**a**) 11 mm, (**c**) 10.5 mm, (**e**) 12 mm, and (**b**,**d**,**f**) correspond to each of the surgeries after 24 h post−surgery.

NTP therapy was applied to open surgical wounds, following a procedure similar to that used for laparoscopic surgeries. After surgery, the deep fascia was ruptured using a polyglycolic acid suture (Vicryl size: 1-0). NTP was then applied with an irradiance of 0.5 W/cm^2^ for four minutes for surgical wounds up to 10 cm in length and five minutes for longer wounds. Subsequently, the superficial fascia was sutured using subdermal points with Prolene size 3-0. Later, NTP was once more administered to the surface wound, utilizing an irradiance of 0.5 W/cm^2^. The outcomes obtained closely resembled those previously outlined. Figure 6a,c,e display two photographs of three different patients after surgery. Additionally, photos taken 24 h post-surgery are shown in Figure 6b,d,f.

It is widely recognized that laparoscopic surgeries offer several advantages over open surgeries, including reduced postoperative pain, a decreased risk of wound infection, reduced hospitalization durations, and a quicker resumption of regular activities. Interestingly, the NTP application demonstrated little difference in wounds from laparoscopic and open surgeries. In both cases, patients were discharged 24 h after surgery, experiencing practically the same level of pain, with no instances of infection or superficial dehiscence.

Effective postoperative pain management minimizes pain-related complications, reduces costs, and promotes early mobilization. According to the results presented in Table 3, the average age for women was 53.23 years, while for men, it was 50.58 years, with a standard deviation (σ) above the average for women at 10.73 years and for men at 10.81 years. In NTP-treated wounds, 77% of women reported mild pain and 23% reported moderate pain, while men reported mild pain in 54% and moderate pain in 46% of cases. It is noteworthy that in surgical wounds treated with NTP, soft pain predominates, accounting for 70.0% of laparoscopic surgeries and 60% of open surgeries. Additionally, women consistently reported less pain than men. In surgeries where NTP was not applied, both male and female patients reported moderate-to-severe pain.

**Figure 7:**
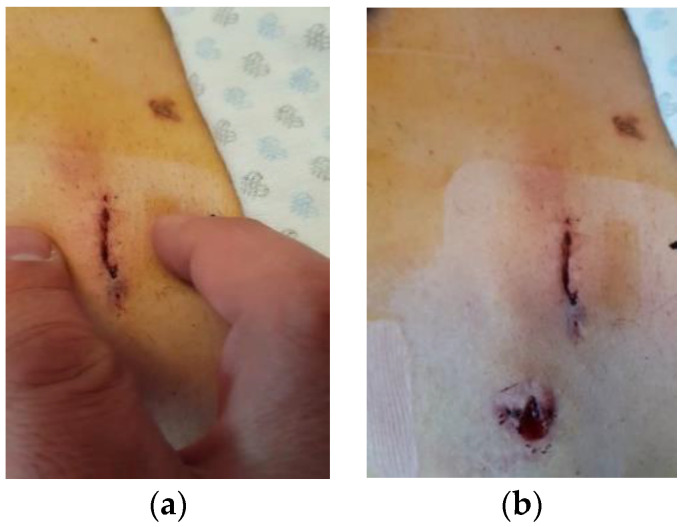
(**a**) Post-surgical wound tension test 24 h later. (**a**) Application of wound lip tension to the wound to which the NTP was applied is shown; (**b**) result after tension test. The upper part corresponds to the wound treated with NTP, which remained intact, while the lower part shows the wound that was not treated with NTP, and it is observed that it suffered damage when subjected to tension.

To compare the benefits of non-thermal plasma (NTP) in the healing of surgical wounds, a test was performed 24 h after the intervention. Tension was applied to the wound’s edges during this test, as shown in Figure 7a. The results revealed that in Figure 7b, where NTP was used, the wound did not show any tissue damage when tension was applied to the ends of the wound lips (upper wound), and where NTP was not applied, it could not withstand the tension exerted (inferior wound). These findings highlight the advantages of NTP in surgical wound closure. By strengthening the wound’s resistance to tension, NTP treatment promotes faster and more successful patient recovery. This effectively improves postoperative outcomes and further validates the effectiveness of NTP at promoting surgical wound healing.

The effectiveness of the NTP procedure was evaluated using the χ^2^ method, and a result of 4.28 was obtained. This value indicated the effectiveness of NTP at promoting faster wound closure. Several scientific reports had reported that laparoscopic and open wound closure using sutures lasted seven to ten days [53,54]. In this study, the suture application results were of the same order. However, a significant difference in closure time was found for patients treated with the NTP technique. Surprisingly, the next day, these patients’ skin wounds closed completely. This was demonstrated by applying a tension test between both edges of the laparoscopic wound. In addition to the above, patients reported no discomfort to touch after the third day, indicating a quick and efficient recovery.

Additionally, it was worth noting that the application cost for NTP treatment was less than five dollars. Therefore, the use of NTP in surgical wounds proves to be not only cost-effective but also highly efficient. Following NTP treatment, patients were clinically able to resume their activities within just three days.

## 4. Discussion

In alignment with the information presented in Section 1, a medical protocol was meticulously developed, gaining prior acceptance and approval from the Health Research and Research Ethics Committees of the ISSEMyM Medical Center. This protocol dictated the use of non-thermal plasma (NTP) to close surgical wounds within the abdominal cavity, covering both laparoscopic and open procedures.

The approved medical protocol was enacted and demonstrated a discernible impact on postoperative tissue in the cohort of fifty patients who willingly provided informed consent for participation in the research project. A comparative assessment of wound healing between suturing and NTP highlighted significant findings.

After NTP was applied, a comparative evaluation revealed that 24 h post-application, the healing progress was equivalent to seven to ten days for conventional suture methods. This observation was further substantiated when patients returned for stitch removal in wounds where NTP was not applied, typically one week after surgery. The accelerated healing process observed in the NTP-treated wounds underscores the promising potential of this innovative approach in enhancing postoperative recovery outcomes.

Despite reports indicating potential postoperative risks in laparoscopic surgeries, such as infection [55], pain [56], pruritus [57], or serohematic drainage [58], and more pronounced risks in open abdominal surgical wounds [59,60,61], the application of NTP in both types of surgeries demonstrated the absence of infection, pain, itching, erythema, or purulent discharge. Notably, no cases of sepsis were reported. This positive outcome may be attributed to the presence of reactive oxygen species (ROS) generated through the interaction of NTP with atmospheric particles in the air [46,47].

Surgical wound dehiscence, a complication seen in some surgeries, including laparoscopic ones [62,63], did not occur in any cases treated with NTP. This notable outcome could be linked to the generation of nitric oxide (NO) (Figure 1), a substance with crucial physiological characteristics that aided wound closure [64].

It is important to note that during and after applying the proposed procedure through the PNT, it never caused thermal damage. Before applying non-thermal plasma (NTP) in patients, we carried out rigorous temperature measurements using a type K thermocouple directly at the tip of the plasma reactor. These measurements were made at different distances, ranging from 1 mm to 10 mm. We observed that at 1 mm, the temperature recorded was 33 °C; at 5 mm, it was 29 °C; and at 10 mm, the temperature was practically the same as the ambient temperature. Therefore, it was essential to gently sweep the NTP over the surface to respect the distance. The increase in temperature in the environment at the distance at which the NTP was applied was, as mentioned, 1–2 °C. The exposure time and maximum irradiance applied was 0.5 W/cm^2^, a magnitude lower than 4.0 W/cm^2^, the maximum permissible value established by the ICNIRP.

Furthermore, it is essential to note that thermal damage to the tissue generally causes an evident inflammatory response, which manifests itself through redness around the wound, excessive discharge, increased pain, changes in the color or size of the wound, and the appearance of red spots on the surrounding skin. However, none of these symptoms were observed in our treated patients. An undesirable situation may occur when surgical wounds are treated with other means, such as a laser [65,66]. As a curious fact, one of the patients had a keloid scar resulting from a previous surgery (Figure 6a,b); however with the application of NTP, the patient did not experience this type of healing again. These findings supported our claim that applying NTP did not cause any thermal damage to the tissues of surgical patients. Our evidence demonstrates that no inflammatory response was activated, further supporting the safety and effectiveness of NTP as a therapeutic method without thermal damage for our patients.

Based on the previous arguments and considering all the cases treated, it was shown that the proposed procedure is safe and effective. In addition, the 50 patients treated with the NTP saw the aesthetics of their wounds improve. This feature indicated that the amount of inflammation after NTP treatment was less than that obtained from conventional wound healing methods [13]. These features of NTP could have been incredibly beneficial for patients.

Considering the evidence presented and the comprehensive analysis of all cases treated, it can be concluded that the proposed NTP procedure was safe and effective [13]. Additionally, the aesthetic improvement observed in the wounds of the 50 treated patients indicates that NTP led to reduced inflammation compared to conventional wound healing methods. These distinctive features of NTP hold considerable potential for providing significant patient benefits.

## 5. Conclusions

It is essential to avoid postoperative complications such as forming small seromas or bruises, dehiscence, wound infection, wound pain, serum accumulation, bleeding, hypertrophic scars, and keloids. Therefore, the surgeon had to employ an optimal strategy for the closure of each wound. The results of this pilot study indicate that there is an alternative procedure, applying a promising technology based on non-thermal plasma produced at atmospheric pressure combined with fascia sutures. This procedure proved to be very effective in treating laparoscopic and open surgical wounds and did not produce fascial dehiscence or postoperative infection. A critical result to highlight is that after applying the procedure to surgical wounds, regardless of their size in the operating room, at twenty-four hours, patients have a healing process equivalent to seven to ten days of typical wound healing. Therefore, it is an excellent result since the utilization of this procedure would reduce patients’ stays in hospital. They would quickly return to productive activities. Also, the NTP procedure is reproducible and efficient. Finally, the patients treated with NTP did not show itching, redness, or discharge from the wound, and pain improved compared to the sutured wounds.

## Figures and Tables

**Table 1:** Laparoscopic surgeries.

	Cholecystectomies	Fundoplications	Appendectomies
Number of patients	14	11	3
Average age (years old)	48 (33–65)	43 (23–69)	52 (38–63)
Female	10 (78.57%)	6 (54.55%)	3 (100%)
Male	4 (21.43%)	5 (45.45%)	0
Duration of surgery (average)	110 min	130 min	65 min
Clinical stay	24 h	24 h	36 h

**Table 2:** Inguinal plasties.

	Laparoscopic	Open
Number of patients	12	10
Average age (years old)	47 (33–65)	58 (49–69)
Female	5 (41.67%)	2 (20%)
Male	7 (58.33%)	8 (80%)
Direct hernias	5 (41.67%)	3 (30%)
Indirect hernias	6 (50%)	4 (40%)
Femoral hernias	1 (8.33%)	1 (10%)
Bilateral hernias		2 (20%)
Wound complications	1 (8.33%)	
Duration of surgery (average)	85 min	55 min
Clinical stay	24 h	24 h

**Table 3:** Postoperative pain.

Laparoscopic Surgeries							
Age	Gender	Number	%	Soft Pain		Moderate Pain	
				(1 to 3)		(4 to 6)	
				Number	%	Number	%
18–30	Female	0	0.0	0	0.0	0	0.0
	Male	1	2.5	0	0.0	1	2.5
31–40	Female	7	17.5	6	15.0	1	2.5
	Male	6	15.0	5	12.5	1	2.5
41–50	Female	4	10.0	4	10.0	0	0.0
	Male	2	5.0	1	2.5	1	2.5
51–60	Female	7	17.5	6	15.0	1	2.5
	Male	4	10.0	2	5.0	2	5.0
>61	Female	6	15.0	2	5.0	4	10.0
	Male	3	7.5	2	5.0	1	2.5
Subtotal	Female	24	60.0	18	45.0	6	15.0
	Male	16	40.0	9	25.0	7	15.0
Total		40	100.0	27	70.0	13	30.0
**Open Surgeries**							
41–50	Female	1	10.0	1	10	0	0
	Male	3	30.0	2	20	1	10
51–60	Female	0	0.0	0	0	0	0
	Male	4	40.0	2	20	2	20
>61	Female	1	10.0	1	10	0	0
	Male	1	10.0	0	0	1	10
Subtotal	Female	2	20.0	2	20	0	0
	Male	8	80.0	4	40	4	40
Total		10	100	6	60	4	40

## Data Availability

We regret to inform you that due to ethical restrictions, we cannot provide specific clinical data for the article.
